# Outcome following mini-open lower limb fasciotomy for chronic exertional compartment syndrome

**DOI:** 10.1007/s00590-021-02919-z

**Published:** 2021-03-06

**Authors:** William M. Oliver, Dominic Rhatigan, Samuel P. Mackenzie, Timothy O. White, Andrew D. Duckworth, Samuel G. Molyneux

**Affiliations:** 1 Edinburgh Orthopaedics, Royal Infirmary of Edinburgh, 51 Little France Crescent, Edinburgh, EH16 4SA UK; 2 Usher Institute, University of Edinburgh, 49 Little France Crescent, Edinburgh, EH16 4SB UK

**Keywords:** Mini-open, Minimally invasive, Lower limb fasciotomy, Exertional compartment syndrome, Patient-reported outcomes, Return to sport

## Abstract

**Purpose:**

The aim of this study was to report outcomes following mini-open lower limb fasciotomy (MLLF) in active adults with chronic exertional compartment syndrome (CECS).

**Methods:**

From 2013–2018, 38 consecutive patients (mean age 31 years [16–60], 71% [*n* = 27/38] male) underwent MLLF. There were 21 unilateral procedures, 10 simultaneous bilateral and 7 staged bilateral. There were 22 anterior fasciotomies, five posterior and 11 four-compartment. Early complications were determined from medical records of 37/38 patients (97%) at a mean of four months (1–19). Patient-reported outcomes (including EuroQol scores [EQ-5D/EQ-VAS], return to sport and satisfaction) were obtained via postal survey from 27/38 respondents (71%) at a mean of 3.7 years (0.3–6.4).

**Results:**

Complications occurred in 16% (*n* = 6/37): superficial infection (11%, *n* = 4/37), deep infection (3%, *n* = 1/37) and wound dehiscence (3%, *n* = 1/37). Eight per cent (*n* = 3/37) required revision fasciotomy for recurrent leg pain. At longer-term follow-up, 30% (*n* = 8/27) were asymptomatic and another 56% (*n* = 15/27) reported improved symptoms. The mean pain score improved from 6.1 to 2.5 during normal activity and 9.1 to 4.7 during sport (both *p* < 0.001). The mean EQ-5D was 0.781 (0.130–1) and EQ-VAS 77 (33–95). Of 25 patients playing sport preoperatively, 64% (*n* = 16/25) returned, 75% (*n* = 12/16) reporting improved exercise tolerance. Seventy-four per cent (*n* = 20/27) were satisfied and 81% (*n* = 22/27) would recommend the procedure.

**Conclusion:**

MLLF is safe and effective for active adults with CECS. The revision rate is low, and although recurrent symptoms are common most achieve symptomatic improvement, with reduced activity-related leg pain and good health-related quality of life. The majority return to sport and are satisfied with their outcome.

## Introduction

Chronic exertional compartment syndrome (CECS) is a condition which accounts for up to 34% of exercise-induced leg pain [1], with an estimated incidence of around 0.5 per 1,000 person-years in active populations [2]. The pathophysiology is thought to involve a reversible increase in intracompartmental pressure during endurance exercise, resulting in transient pain or neurovascular dysfunction [3]. Diagnosis is predominantly based upon the clinical history and exclusion of other more common causes of exertional leg pain (e.g. shin splints), although compartment pressure measurement is often used to confirm the diagnosis [4].

Although non-operative management may be effective in some instances [4], CECS symptoms often persist [5] and lower limb fasciotomy an accepted surgical treatment to facilitate return to activity [6]. Fasciotomy may be performed open [6–19] or using a percutaneous or mini-open approach [20–23]. There is considerable variation in operative technique and reported outcome. Many series focus on younger athletes [12–15, 17, 22–24], military personnel [8, 18] or both [20], despite CECS also occurring in active older populations [25, 26]. Furthermore, the use of validated patient-reported outcome measures (PROMs) is uncommon [8, 9, 23] and we are aware of only one series assessing health-related quality of life after lower limb fasciotomy due to limited use of PROMs in the published literature [23].

The aim of this study was to report outcomes for a consecutive cohort of unselected adults undergoing mini-open lower limb fasciotomy (MLLF) for CECS using a standardised technique. The primary short-term outcome measure was the rate of complications. The primary longer-term outcome measure was patient-reported symptomatic improvement. Secondary longer-term outcome measures included the EuroQol Five-Dimension Three-Level Health Outcome score (EQ-5D) and Visual Analogue Scale (EQ-VAS) [27], return to sport and patient satisfaction.

## Patients and methods

### Study cohort

Patients were retrospectively identified from outpatient records and operating theatre lists (Table 1). Thirty-eight patients were included in the study cohort. The study was assessed by the local research ethics service and registered with the musculoskeletal quality improvement committee.

**Table 1:** Inclusion and exclusion criteria for the study cohort

Inclusion criteria	Exclusion criteria
Adult patients (age ≥ 16 years)	Acute compartment syndrome
Chronic exertional compartment syndrome	Open fasciotomy
Mini-open fasciotomy	
Surgery performed November 2013 to November 2018	

### Cohort characteristics

The mean age was 31 years (range 16–60) and 71% (*n* = 27/38) were male. Ten patients (26%) had documented medical comorbidities. Of patients for whom data regarding smoking and body mass index (BMI) were available (*n* = 27/38), 7% (*n* = 2/27) were smokers and the mean BMI was 29.1 kg/m^2^ (range 21.0–36.0). All patients reported exercise-induced leg pain preoperatively. Nine patients (24%) had unilateral symptoms and 29 (76%) bilateral symptoms. Sensory disturbance was reported by 11 patients (29%), exercise-induced leg swelling by six patients (16%) and subjective weakness by one patient (3%). The mean duration of preoperative symptoms was 4.9 years (range 0.4–42, Table 2). There was not a standardised protocol for non-operative management prior to fasciotomy surgery.

**Table 2:** Patient background and preoperative symptoms for the study cohort (*n* = 38)

*Gender (n, %)*	
Male	27, 71%
Female	11, 29%
*Age at surgery (years)*	
Mean ± SD	31.7 ± 10.7
95% CI	28.2–35.3
Median (range)	30.9 (16.2–60.8)
*Medical comorbidities (n, %)*	
None	28, 74%
≥ 1	10, 26%
*Current smoking status (n, %)*	
Non-smoker	25, 66%
Smoker	2, 5%
Unknown	11, 29%
*Current alcohol intake (n, %)*	
None	10, 37%
Social	16, 59%
Moderate	0
Heavy	1, 3%
Unknown	11, 29%
*BMI (kg/m*^*2*^*)*	
Mean ± SD	29.1 ± 4.8
95% CI	27.3–31.0
Median (range)	29.1 (21.0–36.0)
*Obesity status (n, %)*	
Non-obese	15, 40%
Obese	12, 32%
Unknown	11, 29%
*SIMD Quintile (n, %)*	
1 (most deprived)	2, 5%
2	12, 32%
3	6, 16%
4	7, 18%
5 (least deprived)	11, 29%
*Affected side (n, %)*	
Right	5, 13%
Left	4, 11%
Bilateral	29, 76%
*Preoperative pain (n, %)*	
No	0
Yes	38, 100%
*Preoperative swelling (n, %)*	
No	32, 84%
Yes	6, 16%
*Preoperative motor disturbance (n, %)*	
No	37, 97%
Yes	1, 3%
*Preoperative sensory disturbance (n, %)*	
No	27, 71%
Yes	11, 29%
*Preoperative symptom duration (months)*	
Mean ± SD	59.3 ± 91.4
95% CI	28.0–90.7
Median (range)	31 (5–504)
*Side of surgery (n, %)*	
Unilateral	21, 55%
Simultaneous bilateral	10, 26%
Staged bilateral	7, 18%
*Interval between staged bilateral procedures (weeks)*	
Mean ± SD	15.8 ± 10.4
95% CI	7.1–24.5
Median (range)	14.5 (3–35.1)
*Compartments released (n, %)*	
Anterolateral only	22, 58%
Posterior only	5, 13%
Four-compartment	11, 29%

BMI, body mass index; CI, confidence interval; SD, standard deviation; SIMD, Scottish Index of Multiple Deprivation

NB. Scottish Index of Multiple Deprivation assigned according to postcode at the time of surgery (see Scottish Government Scottish Index of Multiple Deprivation: SIMD16 Technical Notes. https://www2.gov.scot/Resource/0050/00504822.pdf)

Of patients for whom complete data regarding preoperative sporting participation were available (*n* = 27/38), 93% (*n* = 25/27) were involved in sport prior to symptom onset, 44% (*n* = 12/27) playing socially/recreationally and 48% (*n* = 13/27) playing competitively. However, by the time of surgery, 56% (*n* = 15/27) were no longer participating in sport as a result of their symptoms, leaving only 37% (*n* = 10/27) involved in sport (social/recreational 22%, *n* = 6/27; competitive 15%, *n* = 4/27).

### Compartment pressure measurement

Thirty-one patients (82%) underwent preoperative compartment pressure measurement (CPM). The remaining seven patients gave a clear and typical history of CECS (transient, deep-seated muscular pain during exercise, occurring at a predictable and reproducible timepoint, in the absence of underlying vascular disease), and CPM was not considered necessary to confirm the diagnosis. To exclude alternative diagnoses, five of these seven patients underwent other imaging studies as part of their preoperative work-up, including plain radiographs (*n* = 4), MRI (*n* = 3) and arterial Doppler ultrasound (*n* = 1).

**Fig. 1:**
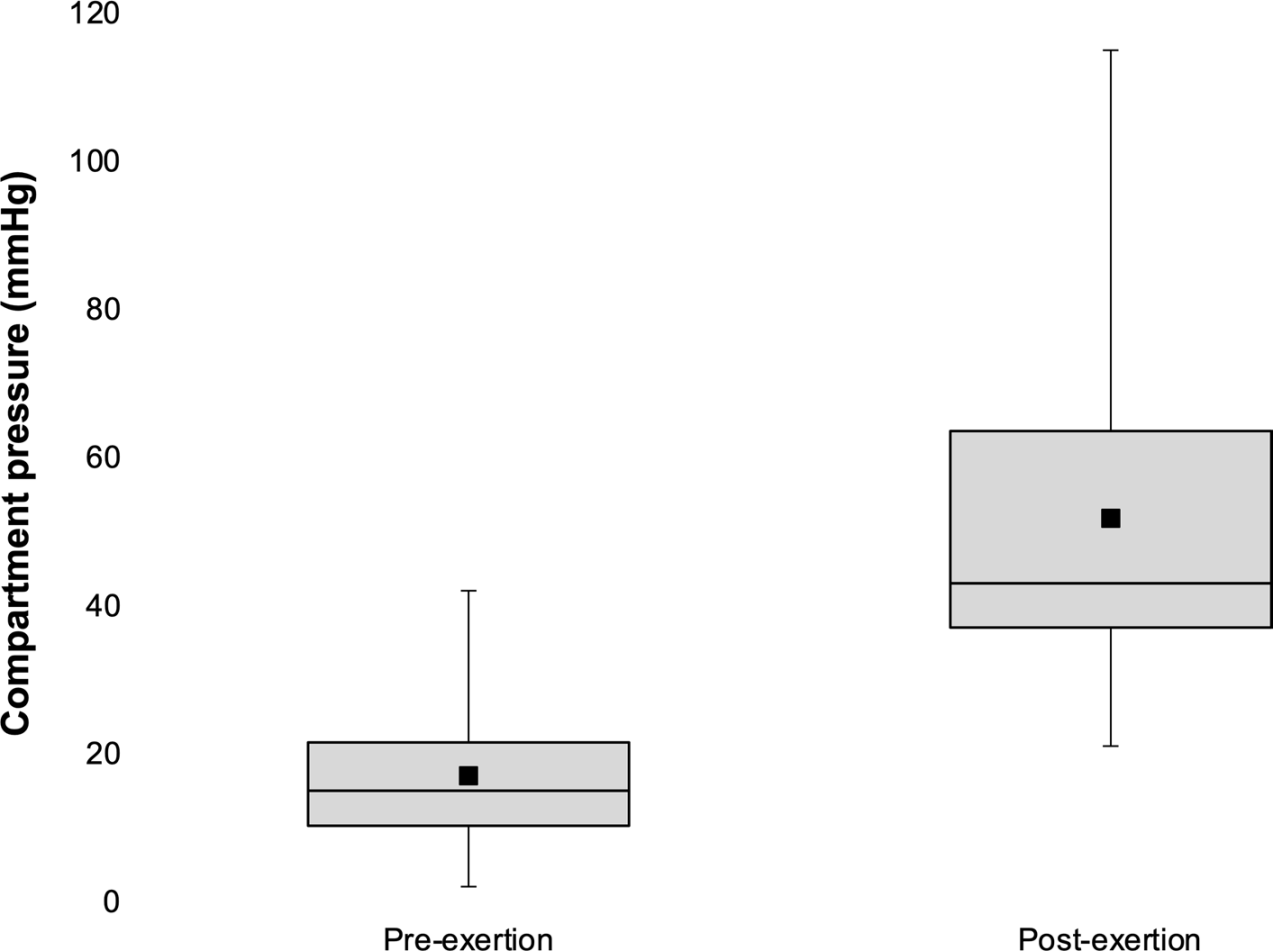
Box-and-whisker plot showing leg compartment pressure measurements, pre- and post-exertion (*n* = 31); black square represents the mean value

For 31 patients, CPM was performed in the outpatient department under local anaesthesia with a monitor inserted into the anterior compartment of the leg using a slit-catheter technique [28]. In patients with bilateral symptoms, the more symptomatic leg was measured. The mean pre-exertion pressure was 17 mmHg (range 2–42). Patients then underwent a standardised exercise protocol on a treadmill. Treadmill speed was initially set between 5.0 and 12.0 km/h (depending on the patient’s reported symptoms and level of fitness) with an incline of 2%. Treadmill speed and incline were increased every minute (by 1.0 km/h and 1%, respectively), until the patient described their leg pain as severe or intolerable. Post-exertion compartment pressures were measured one minute following exercise (mean 52 mmHg, range 21–115; Fig. 1). For study purposes, CPM was considered ‘positive’ with either a pre-exertion pressure ≥ 15 mmHg or a one-minute post-exertion pressure ≥ 30 mmHg [1]; 29 patients (94%) had positive CPMs based upon these criteria. The two patients with ‘negative’ CPMs nonetheless demonstrated an increase in compartment pressure (from 2 to 21 mmHg and from 10 to 26 mmHg), accompanied by convincing symptoms during testing. Both patients opted to proceed to MLLF after an informed discussion with the treating surgeon.

### Surgical intervention

All patients underwent MLLF using a standardised technique under the care of a single surgeon. Surgery was performed under general anaesthesia, with the patient supine and a thigh tourniquet applied.

**Fig. 2:**
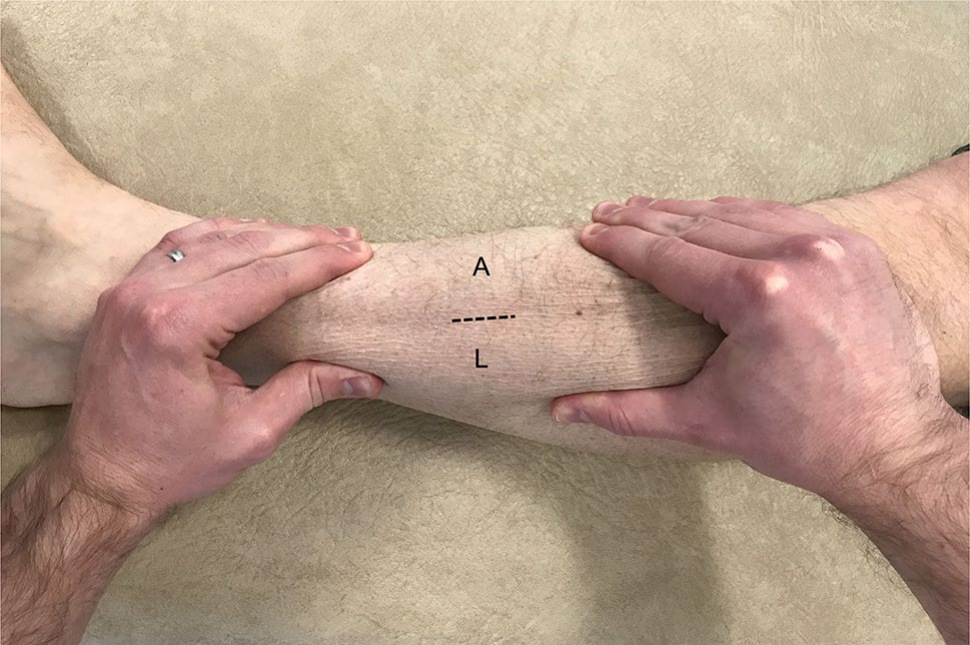
Lateral aspect of the left leg. The surgeon performs the ‘squeeze test’ to identify the insertion of anterior intermuscular septum onto the deep fascia; this marks the location of a 2.5 cm longitudinal incision (dotted line) for mini-open fasciotomy of the anterior (A) and lateral (L) compartments

For decompression of the anterior and lateral compartments, a 2.5 cm longitudinal incision was made over the anterior intermuscular septum (AIS), which was identified using the ‘squeeze test’ (Fig. 2). In obese patients, the incision was made two finger breadths lateral to the anterior tibial crest. The incision was centred over the area of greatest muscle bulk. Following sharp dissection of subcutaneous tissues, the deep fascia was identified and the overlying soft tissues retracted, in order to visualise the AIS as a white stripe thickening in the fascial layer. A 1 cm horizontal incision was used to confirm the position of the AIS. The soft tissues overlying the fascia were cleared distally and proximally using Mayo scissors (inserted closed and extracted open). Langenbeck retractors were used to elevate the soft tissues, while the fascia of each compartment was released longitudinally (distally then proximally) along its entire length, using a firm sweep with partially closed Metzenbaum scissors, the curve pointing away from the AIS. Care was taken to ensure the fascia was released as far as the musculotendinous junction in both directions; distally this necessitated a short area of blind dissection. Adequate release was confirmed visually and digitally.

For the posterior compartments, an incision was made one finger-breadth medial to the medial tibial crest, centred over the maximal muscle bulk. This incision was between 4 and 10 cm in length, as required to allow visualisation of the prominent venous network in this area. The superficial posterior compartment fascia was incised with a scalpel 1 cm posterior to the posteromedial border of the tibia. Proximal and distal fasciotomy were completed along the entire length of the fascia using Metzenbaum scissors, as above. The deep posterior compartment fascia was identified through the superficial compartment fasciotomy and completed in the same way.

The tourniquet was released prior to closure to allow meticulous haemostasis. Skin incisions were closed with absorbable subcuticular suture and steristrips, and an adhesive dressing and compressive crepe bandage applied. Patients were allowed full, active range of motion and full weight-bearing as tolerated. There was no formal post-operative rehabilitation protocol. Outpatient review was performed at twelve weeks post-operatively, with further reviews as clinically indicated.

A unilateral procedure was undertaken in 21 patients, simultaneous bilateral procedures in 10 patients and staged bilateral procedures in seven patients. Given the difficulties with post-operative rehabilitation following simultaneous bilateral procedures, our default strategy was to offer staged bilateral procedures to patients with bilateral symptoms. However, where patients requested expedited surgical intervention and this was considered practical, simultaneous bilateral procedures were offered. Of 29 patients with bilateral symptoms, seven underwent staged bilateral procedures and 10 underwent simultaneous bilateral procedures. The remaining 12 patients underwent unilateral surgery, electing not to proceed to a staged contralateral procedure. Isolated anterolateral compartment fasciotomies were performed in 22 patients, isolated posterior compartment fasciotomies in five patients and four-compartment fasciotomy in 11 patients (Table 2).

### Outcome assessment

Short-term outcomes were retrospectively determined from outpatient records. Specific complications were recorded, including infection, wound dehiscence, recurrent symptoms and the need for revision surgery. Longer-term patient-reported outcomes were obtained via postal survey. Participants were asked to rate preoperative pain during normal and sporting activity using a visual analogue scale (VAS; 0 = no pain, 10 = worst pain imaginable). Details regarding post-operative symptom recurrence and severity were recorded. Current symptoms, including pain during normal activity/sport (as above), abnormal sensation at the site of their surgery (none, only during sport, during any activity, constant) and scar satisfaction (0 = terrible scar, 10 = excellent scar), were documented. Quality of life was assessed using the EuroQol Five-Dimension Three-Level Health Outcome score (EQ-5D) and Visual Analogue Scale (EQ-VAS) [27]. Participants were asked whether they had returned to their main sport post-operatively, and the time taken to do this (within six weeks, between six weeks and three months, between three and six months, between six and 12 months). Finally, participants were asked to indicate satisfaction with their outcome (very satisfied, satisfied, neutral, dissatisfied, very dissatisfied) and how likely they would be to recommend the procedure to others (very likely, likely, neutral, unlikely, very unlikely).

### Statistical methods

Statistical analysis was performed using SPSS version 23.0 (IBM, Armonk, NY). The difference between pre- and post-operative pain scores was assessed using the Wilcoxon signed-rank test. Significance was set at *p* < 0.05.

## Results

### *Short-term outcomes (n* = *37)*

One patient (3%) did not attend follow-up post-operatively and was excluded. At a mean of four months (range 1–19), complications occurred in six patients (16%), including superficial infection (11%, *n* = 4/37), deep infection (3%, *n* = 1/37) and partial wound dehiscence (3%, *n* = 1/37). No instances of post-operative peroneal nerve palsy were documented and no patient required further operative intervention for surgical complications.

Recurrent symptoms were documented in 14 patients (38%), including leg pain (30%, *n* = 11/37), sensory disturbance (11%, *n* = 4/37) and subjective muscle weakness (3%, *n* = 1/37). Three patients (8%) required a revision fasciotomy for recurrent exercise-induced leg pain (Table 3).

**Table 3:** Summary of revision procedures following mini-open lower limb fasciotomy for the short-term follow-up cohort (*n* = 37)

Gender, age (years)	Primary procedure	Revision indication	Time of revision (months)	Revision procedure
Male, 25	Simultaneous bilateral four-compartment	Pain	6.4, 21	Staged bilateral open posterior compartment
Female, 31	Unilateral four-compartment	Pain	16	Unilateral open four-compartment
Female, 26	Simultaneous bilateral four-compartment	Pain	27	Bilateral open posterior compartment

### *Longer-term outcomes (n* = *27)*

Six patients (16%) declined to complete the postal survey and five (13%) had moved out-of-region and were uncontactable; the remaining 27 (71%) completed the survey. At a mean of 3.7 years (range 0.3–6.4), eight patients (30%) were asymptomatic. Nineteen patients (70%) had recurrent symptoms, but with 15 (56%) reporting symptomatic improvement, three (11%) reporting no change and only one whose symptoms were worse post-operatively. Twenty patients (74%) were satisfied or very satisfied following their procedure, five (19%) were neutral and two (7%) were dissatisfied. Twenty-two patients (81%) were likely or very likely to recommend the procedure, four (15%) were neutral and one (4%) was unlikely to do so.

**Fig. 3:**
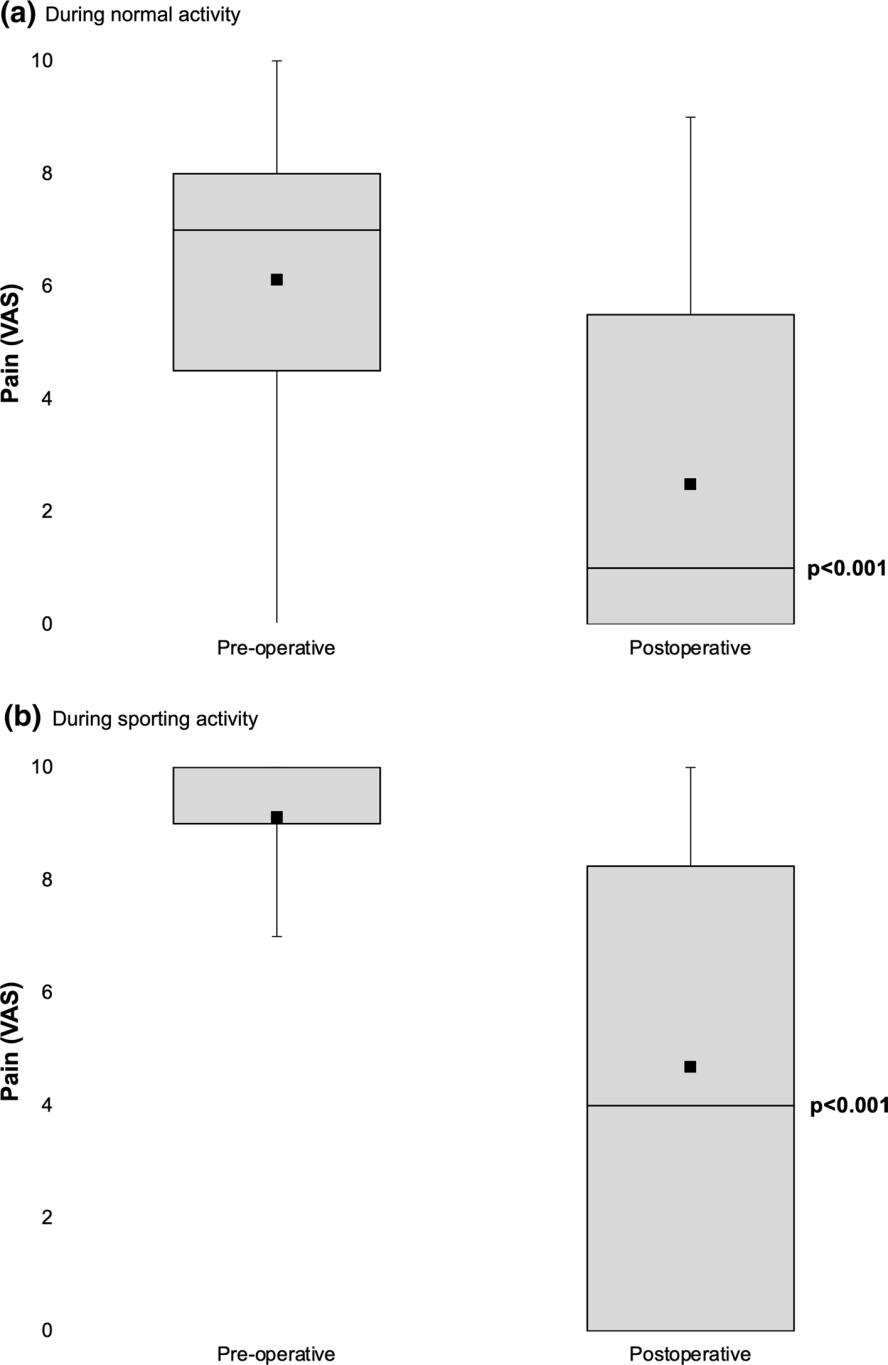
Box-and-whisker plots showing pain (visual analogue scale), before and after mini-open lower limb fasciotomy (*n* = 27); black square represents the mean value. **(a)** During normal activity. **(b)** During sporting activity

**Fig. 4:**
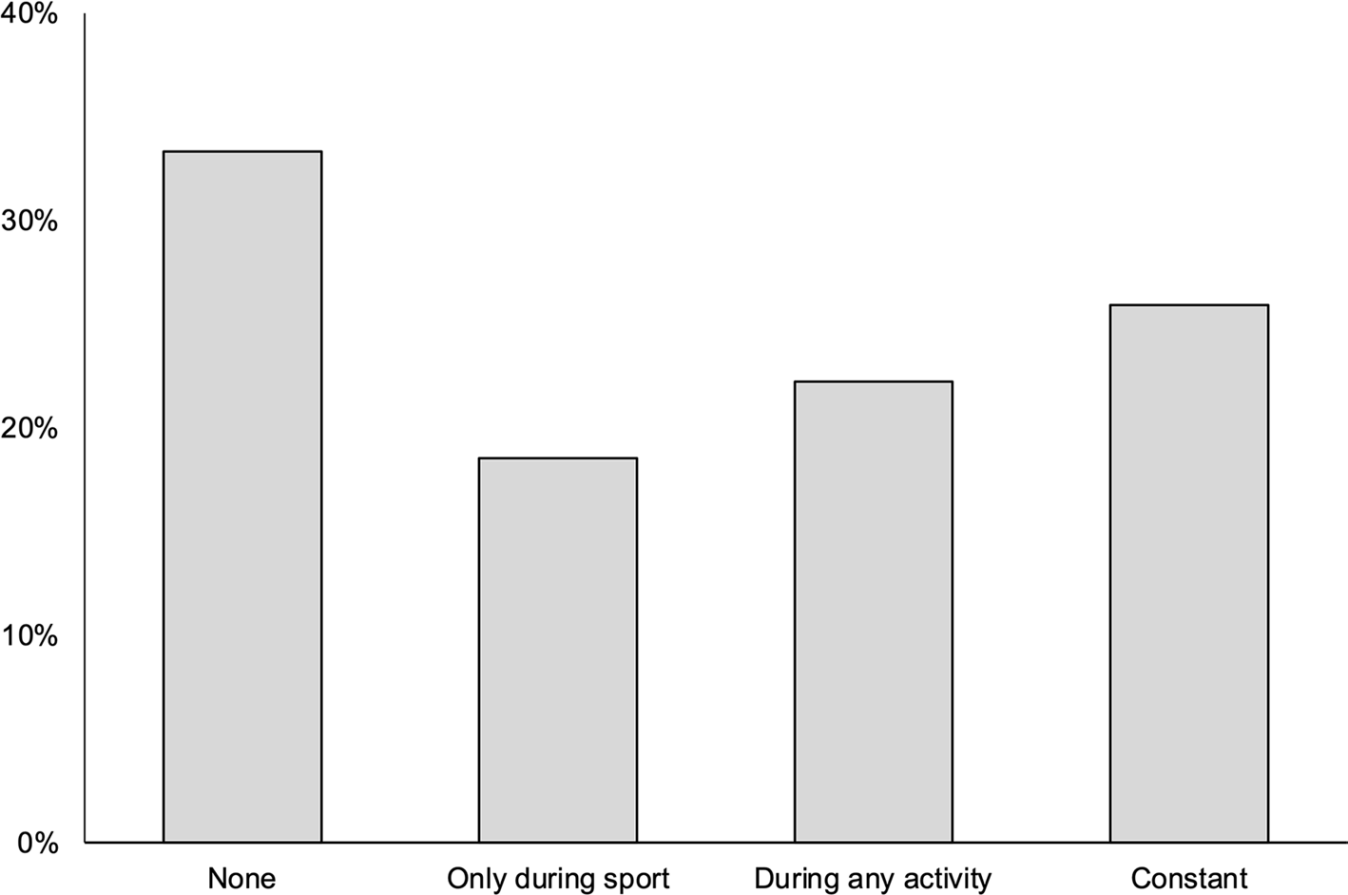
Patient-reported abnormal sensation at the site of surgery following mini-open lower limb fasciotomy (*n* = 27)

Eighteen patients (67%) reported recurrent pain post-operatively. However, mean pain VAS during normal activity improved from 6.1 to 2.5 (*p* < 0.001), and during sport from 9.1 to 4.7 (*p* < 0.001; Fig. 3). Eighteen patients (67%) reported abnormal sensation at the site of their surgery (Fig. 4). The mean scar satisfaction was 8.2 (range 4–10). The mean EQ-5D was 0.781 (range 0.130–1) and EQ-VAS 77 (range 33–95).

**Fig. 5:**
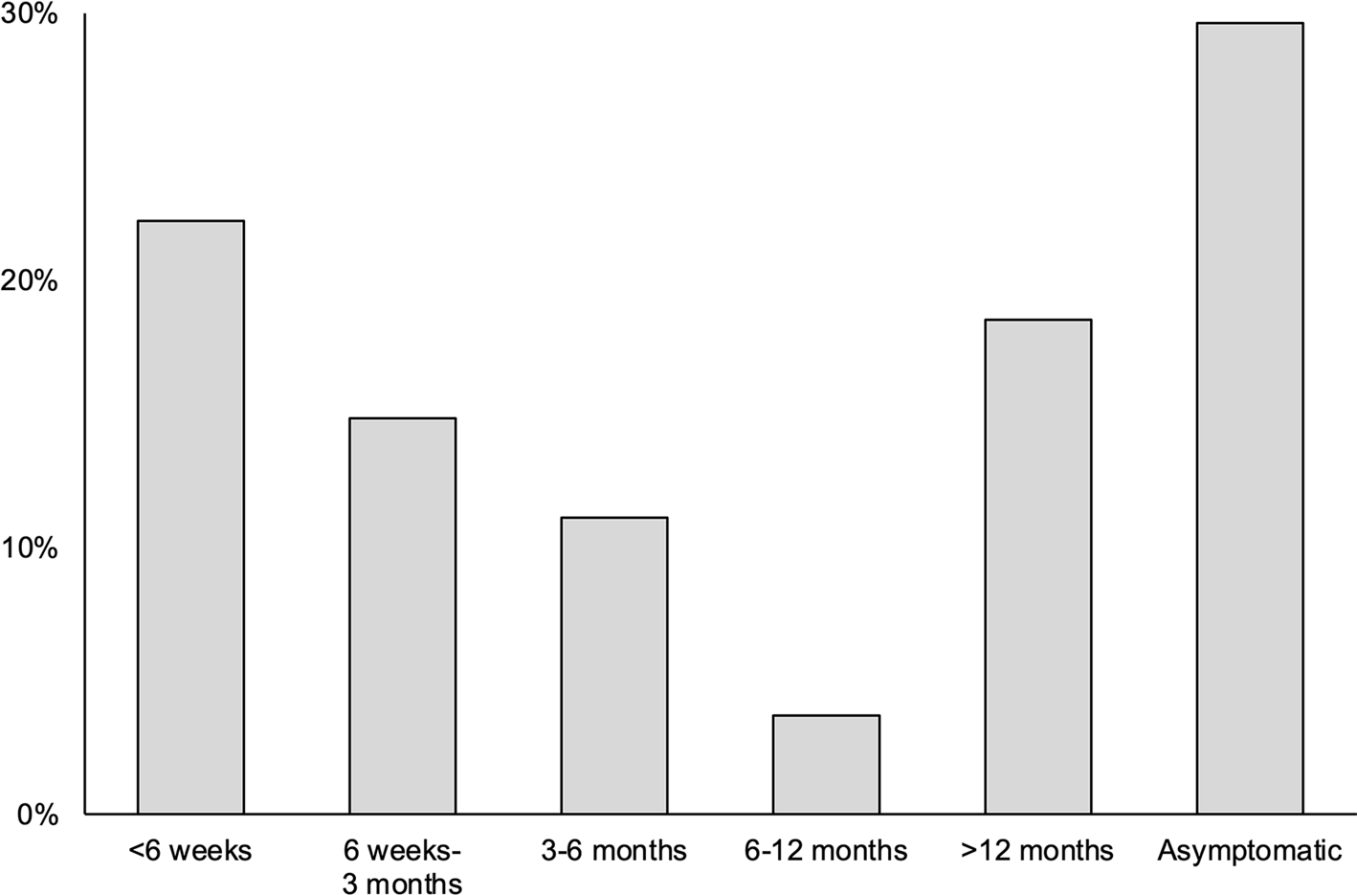
Onset of recurrent symptoms following mini-open lower limb fasciotomy (*n* = 27)

The time at which symptoms returned post-operatively was bimodal (Fig. 5). Of the six patients reporting recurrent symptoms within six weeks, four had positive preoperative CPMs, one had negative CPMs and one had not undergone preoperative CPM. None of the six patients with early recurrence underwent a revision fasciotomy.

**Fig. 6:**
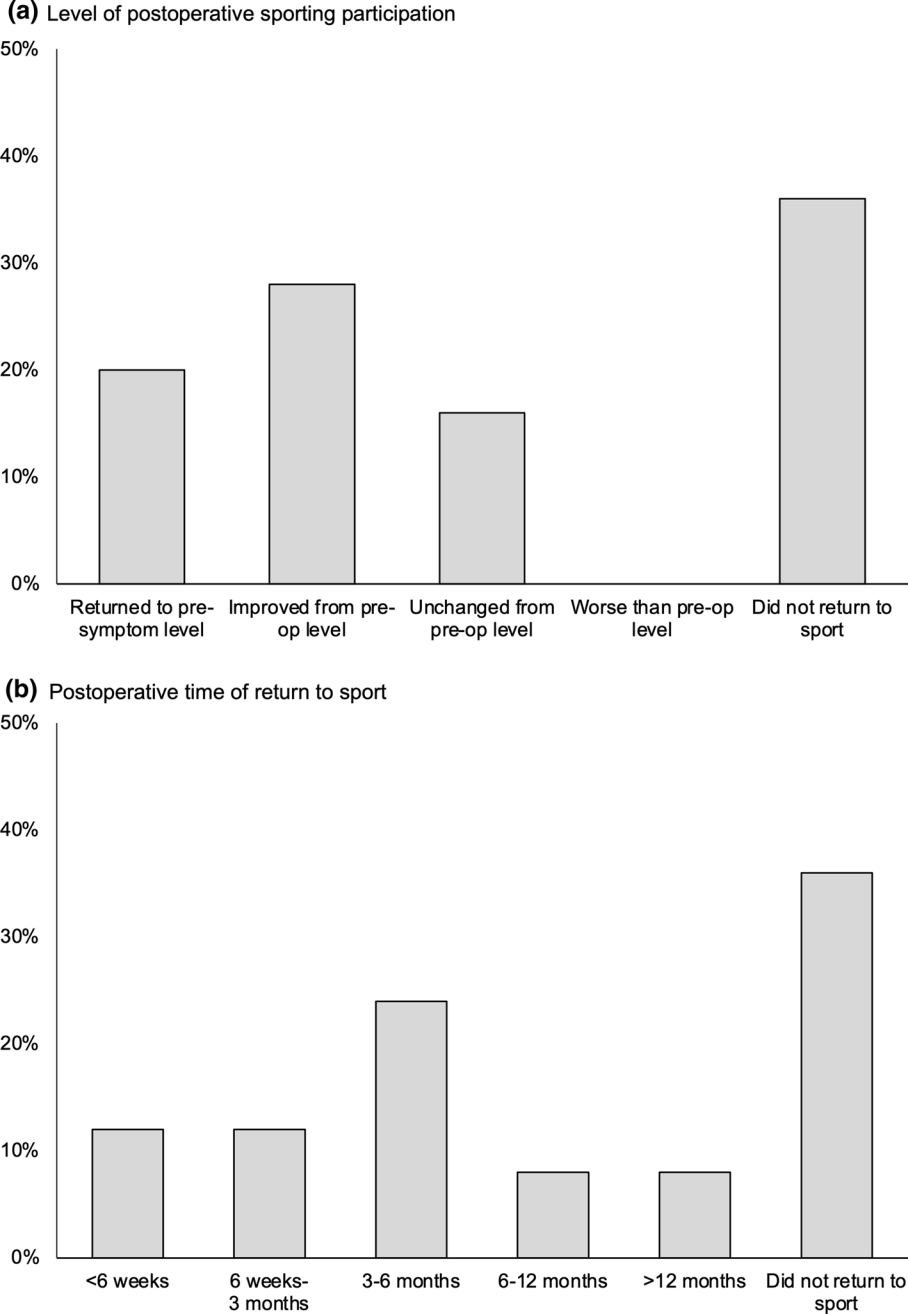
Return to sport following mini-open lower limb fasciotomy (*n* = 27). **(a)** Level of post-operative sporting participation. **(b)** Post-operative time of return to sport

Of patients participating in sport preoperatively (*n* = 25), 16 (64%) returned to their main sport. Five (31%) reached their pre-symptom level of participation, seven (44%) were better than preoperatively (but did not reach their pre-symptom sporting level), and four (25%) were unchanged. Seventy-five per cent (*n* = 12/16) returned to sport within six months (Fig. 6).

## Discussion

This study documents patient-reported outcomes following MLLF in an unselected consecutive cohort of active adult patients. Despite the modest rate of complications, no patient required early secondary surgical intervention. Three patients underwent revision surgery for recurrent leg pain following their primary procedure. Although recurrent symptoms increased over time, 85% (*n* = 23/27) reported a sustained symptomatic improvement in the longer term. Overall, leg pain improved during both normal activity and sport, and around two-thirds of patients returned to sport post-operatively, three-quarters at an improved level of participation and within six months of surgery.

The rate of complications in our study (16%) is similar to other series reporting on both open [6–8, 10, 18] and percutaneous [21, 23] procedures (11–21%). Similarly, our rate of revision surgery is comparable to existing studies documenting reoperation rates between 5% and 11% [7, 9, 10, 16–18], although some report lower rates [21–23]. It has been suggested that the development of recurrent symptoms after fasciotomy could be time-dependent, and that shorter follow-up may underestimate symptom recurrence [9]. Our findings appear to support this observation, although this may reflect a difference between clinicians and patients in expectations of early post-operative recovery and the perceived significance of certain symptoms. At longer-term follow-up, patient-reported return of symptoms was bimodal and most commonly occurred within six weeks of surgery and then beyond 12 months post-operatively. Early recurrence may be due to inadequate decompression, although no patients reporting recurrent symptoms within six weeks went on to a revision procedure. All patients had a history of reproducible exertional leg pain preoperatively, but only four of the six reporting early recurrence had ‘positive’ CPMs [1]; one did not meet the diagnostic criteria (with a pressure increase from 2 to 21 mmHg), and another did not undergo preoperative CPM. The later peak in recurrent symptoms, following an initial symptom-free period, may be due to internal scarring resulting in secondary compartmental constriction [7, 29]. This is consistent with an animal study, which demonstrated that releasing ≥ 90% of the fascial compartment may mitigate the future impact of post-operative scarring [30].

Despite the prevalence of recurrent symptoms, 85% reported a sustained symptomatic improvement compared with their preoperative level. Although the MLLF technique appears to be effective in reducing pain, most patients reported some degree of leg pain at longer-term follow-up, in keeping with existing studies [6, 13, 14]. Abnormal sensation complicates up to 62% of fasciotomy procedures [6, 8, 10, 13–21, 23], and the reasons for the slightly higher prevalence of sensory abnormalities we observed (67%) are unclear. Notably, only one-quarter of patients described constant sensory symptoms, with forty per cent reporting these only on activity. The former may suggest an intraoperative nerve injury, while the latter may be more in keeping with sensory symptoms of recurrent CECS. In either case, our findings have implications for counselling patients considering MLLF; while the majority do derive longer-term benefit, preoperative symptoms may recur in up to two-thirds of patients and sensory disturbance appears to be relatively common.

Health-related quality of life following MLLF was comparable to age-matched populations [31]. Maffulli et al. [23] reported an improvement in EQ-VAS following anterolateral compartment fasciotomy using a two-incision technique. The superior EQ-VAS observed in their study may be due to their younger patient cohort (median age 27, range 18–35) or the fact the posterior compartment was not involved. Similarly, although rates of return to sport appear to be higher among athletes undergoing fasciotomy (75–100% [12–15, 17, 22–24]) compared with more heterogenous groups (76–90% [6, 9, 10]), all existing studies involve younger cohorts than the present study. Older adults with CECS should be counselled that the expected rates of return to sport may not be as high as in younger, athletic populations.

We acknowledge this study is limited by its retrospective design. Despite good short and longer-term retention rates, loss to follow-up may have restricted our ability to determine factors associated with outcome. Preoperative symptoms were obtained from contemporaneous outpatient records, but preoperative pain scores were recorded at longer-term follow-up and were susceptible to recall bias. Obtaining some desirable baseline data, particularly preoperative patient-reported outcomes, was not possible in the context of this retrospective study. Not all patients underwent preoperative CPM, however, we feel this reflects everyday clinical practice, in which a typical history (in the absence of clinical signs suggesting alternative diagnoses) can be sufficient to make the diagnosis. It was not possible to explore the reasons why patients with bilateral symptoms elected not to undergo staged contralateral-sided surgery following their index procedure (*n* = 12), although this may be an avenue for further qualitative research. Future prospective randomised studies should aim to compare outcomes following minimally invasive techniques with traditional open procedures.

## Conclusions

MLLF is safe and effective for active adults with CECS. The rate of complications and revision procedures is comparable to the existing literature. Recurrent symptoms are common, but most patients report symptomatic improvement and a reduction in activity-related leg pain in the longer term. The majority successfully return to sport post-operatively, report good health-related quality of life and are satisfied with their outcome.

## Data Availability

Study data will be made available upon request.
